# Divergence in larval jaw gene expression reflects differential trophic adaptation in haplochromine cichlids prior to foraging

**DOI:** 10.1186/s12862-019-1483-3

**Published:** 2019-07-24

**Authors:** Ehsan Pashay Ahi, Pooja Singh, Anna Duenser, Wolfgang Gessl, Christian Sturmbauer

**Affiliations:** 1 Institute of Biology, University of Graz, Universitätsplatz 2, A-8010 Graz, Austria; 2 Evolutionary Biology Centre, Uppsala University, Norbyvägen 18A, 75236 Uppsala, Sweden

**Keywords:** Haplochromine cichlids, Modularity, jaw development, Trophic specialization, Adaptive radiation, East African lakes

## Abstract

**Background:**

Understanding how variation in gene expression contributes to morphological diversity is a major goal in evolutionary biology. Cichlid fishes from the East African Great lakes exhibit striking diversity in trophic adaptations predicated on the functional modularity of their two sets of jaws (oral and pharyngeal). However, the transcriptional basis of this modularity is not so well understood, as no studies thus far have directly compared the expression of genes in the oral and pharyngeal jaws. Nor is it well understood how gene expression may have contributed to the parallel evolution of trophic morphologies across the replicate cichlid adaptive radiations in Lake Tanganyika, Malawi and Victoria.

**Results:**

We set out to investigate the role of gene expression divergence in cichlid fishes from these three lakes adapted to herbivorous and carnivorous trophic niches. We focused on the development stage prior to the onset of exogenous feeding that is critical for understanding patterns of gene expression after oral and pharyngeal jaw skeletogenesis, anticipating environmental cues. This framework permitted us for the first time to test for signatures of gene expression underlying jaw modularity in convergent eco-morphologies across three independent adaptive radiations. We validated a set of reference genes, with stable expression between the two jaw types and across species, which can be important for future studies of gene expression in cichlid jaws. Next we found evidence of modular and non-modular gene expression between the two jaws, across different trophic niches and lakes. For instance, *prdm1a*, a skeletogenic gene with modular anterior-posterior expression, displayed higher pharyngeal jaw expression and modular expression pattern only in carnivorous species. Furthermore, we found the expression of genes in cichlids jaws from the youngest Lake Victoria to exhibit low modularity compared to the older lakes.

**Conclusion:**

Overall, our results provide cross-species transcriptional comparisons of modularly-regulated skeletogenic genes in the two jaw types, implicating expression differences which might contribute to the formation of divergent trophic morphologies at the stage of larval independence prior to foraging.

**Electronic supplementary material:**

The online version of this article (10.1186/s12862-019-1483-3) contains supplementary material, which is available to authorized users.

## Background

The evolution of jaws in ancestral vertebrates was pivotal in their subsequent colonisation of trophic niches [1]. Jaws arose via modification of particular gill arches [2] and over time, the vertebrate craniofacial anatomy became one of the most complex and modular muscuskeletal systems, with myriad distinct anatomies. The diversity of these anatomies and the trophic adaptations they enabled have fascinated evolutionary biologists for decades and are a major focus of studies on evolutionary novelty [3] as trophic morphology was found to be a strong speciation trait in several diversifying lineages [4–7], including the hyperdiverse cichlid fishes from the East African Great Lakes [8].

Cichlids are one of the most striking examples of trophic diversity as they have evolved highly specialized pharyngeal jaws in addition to oral jaws [9]. The functional decoupling of the oral and pharyngeal jaws is considered a key innovation that catalysed adaptive radiation by allowing the two jaws to evolve independently, thereby boosting versatility in feeding modes. This rapid trophic diversification is thought to have been facilitated by the phenotypically plastic jaws of cichlids [10], allowing for genetic accommodation as proposed by the Flexible Stem Hypothesis [11]. Not only have cichlids adapted to a wide range of feeding methods such as algae browsing/grazing, insect sucking, snail-crushing, fish-scale biting, and fish fry eating and more, but these eco-morphologies have evolved in parallel across different radiations [12, 13]. This makes cichlids an unparalleled model to study divergent trophic morphologies and particularly the pharyngeal jaw apparatus, which is a key feature of these fish. The majority of studies that have explored the functional, morphological, or genetic aspects of cichlids trophic adaption have focused either on the oral or pharyngeal jaw [14–19]. This is especially true for studies of gene expression underlying cichlid trophic specialization. Gene expression is an important determinant of morphological evolution and previous studies have identified suites of genes underlying different diets [16] and diet plasticity [17, 20]. But there is still a dearth of knowledge on direct comparisons of the two cichlid jaws at the transcriptional level with regards to development, growth, morphogenesis, and evolvability. If we are to address questions regarding the molecular basis of their functional modularity and independent evolution from ancestral pharyngeal arches, then we need to study the two units simultaneously.

One ecologically important key stage of cichlid development is the stage 26 defined in [21]. This stage marks the end of larval development with the complete absorption of the yolk sac into the body cavity of the fish larvae. For the mouthbrooding haplochromine cichlids from Lake Tanganyika, Malawi and Victoria, this is the point where in nature the larvae leave the buccal cavity of the mother and begin to forage independently. We have previously shown that at this stage the jaws of species adapted to different trophic niches are morphologically and transcriptionally distinct [16] and ossification of jaw elements has been almost completed or reached a steady-state (unpublished data). Thus, this stage is critical to understand the pattern of gene expression upon completion of oral and pharyngeal jaw skeletogenesis, immediately before exposure to environmental cues. This will allow us to establish whether distinct molecular factors play a predisposing role in differentially adapted species in either of the two jaws, how they are organized in a modular manner (i.e. different between the two jaws), and how later in life they might canalize plastic responses to alternative feeding habits and diet. Insight gained from this will thus not only enhance our understanding of the factors regulating oral and pharyngeal jaw morphology and modularity in a steady-state stage, but also subsequent phenotypic plasticity. It has been proposed that mutations affecting the expression of key genetic factors can have tremendous effects on their downstream gene modules [22]. Such transcriptional changes across generation can potentially lead to adaptive phenotypic trajectories which might be highly responsive (or non-responsive) to environmental stimuli, and thus, distinct in phenotypic plasticity (reviewed in [22]).

In this study, we investigate the expression of a set of modularly regulated genes in the oral and pharyngeal jaws of 12 haplochromine cichlid fish species at stage 26, the end of larval development and prior to the onset of exogenous feeding. The selected candidate genes are known to be involved in the development and morphogenesis of jaw skeletal elements in teleost fishes and also have modular effects along the anterior-posterior axis during viscerocranial skeletogenesis (Table 1). The species cover two major trophic niches in the three Great East African Lakes, i.e. Lake Tanganyika (LT), Lake Malawi (LM) and Lake Victoria (LV). This framework allows us for the first time to test for signatures of gene expression in convergent eco-morphologies across three independent adaptive radiations. Our results provide cross-species expression comparisons of skeletal related genes in the two jaw types at late larval stage in haplochromine cichlids and implicate expression differences by which formation of distinct trophic skeletal morphologies can be determined prior to initiation of plastic molecular responses emanating from contrasting environmental influences and diets.

**Table 1:** Selected target genes involved in the development and morphogenesis of jaw skeletal elements and with modular effects along the anterior-posterior axis during viscerocranial skeletogenesis

Gene	Function(s) at early developmental stages	Modular viscerocranium expression	Potential functions(s) at late developmental stages	References
*bapx1*	Positional specification of oral jaw joint	Anterior	Mediating the effects of growth and morphogenic signals on oral jaw skeletal elements	[23–25]
*foxq1b*	Formation of lower oral jaw	Anterior	Mediating the effects of signals induced by environmental compounds on oral jaw skeletal elements	[26–28]
*wnt9b*	Dorso-ventral patterning of oral jaw	Anterior	Mediating Wnt pathway dependent outgrowth of upper jaw	[29, 30]
*satb2*	Determining length of distal jaw module	Anterior or posterior	Contributing to variations and evolvability of the distal jaw domain	[31, 32]
*fgf8a*	Cell migration towards pharyngeal pouches	Posterior	Determining skeletal size in posterior pharyngeal arches	[33–35]
*lbh*	Morphological variations along anterior-posterior jaw skeleton	Anterior or posterior	Activation of Ap-1 complex, a mechanically induced signal affecting skeletogenesis	[17, 19, 36–38]
*prdm1a*	Patterning and morphogenesis of posterior jaw skeleton	Posterior	Mediating the effects of RA signal on jaw skeleton in response to environmental stimuli (e.g. different diets)	[37, 39–42]

## Results

### Identification of suitable reference genes for qPCR expression analysis

**Fig. 1:**
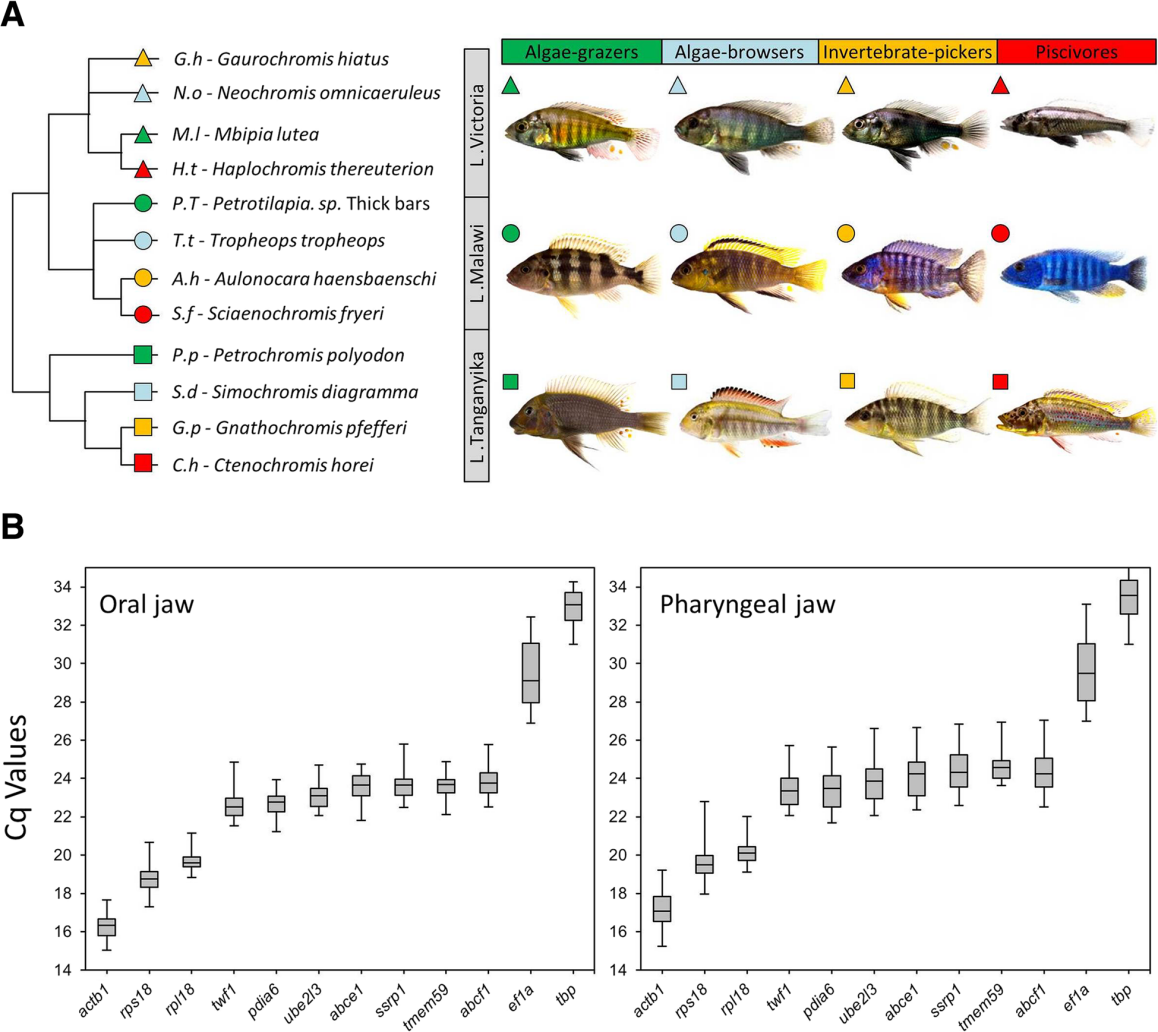
Relatedness, habitats, trophic specialization and expression levels of candidate reference genes in the jaws of haplochromine cichlid species used in this study. **a** A simplified phylogenetic tree of the East African haplochromine cichlids displaying the relatedness between the species specified by their habitats/lakes and trophic specializations. The symbol colour for each species represents related trophic niche whereas the symbol shape refers to its habitat/lake. **b** Expression levels of candidate reference genes based on raw Cq values in oral or pharyngeal jaws across all of the species. In each box plot, the middle line represents the median and boxes lower and upper limits indicate the 25/75 percentiles

To measure the expression of our selected target genes in oral and pharyngeal jaws, it was essential to first validate reference gene(s) with least expression variation among the jaw samples across different species [43]. The 12 candidates were selected from validated reference genes used in studies of different tissues in East African cichlids [19, 44–47], as well as several highly expressed genes without species-specific expression differences from transcriptome data of tissues containing oral and pharyngeal jaws in three haplochromine species (Singh et al. submitted). The candidate reference genes had a range of expression levels, and interestingly, showing very similar expression level patterns between the oral and pharyngeal jaws; from highest levels for *actb1*, *rps18* and *rpl18* to lowest levels for *tbp* and *ef1a*, respectively (Fig. 1b). This indicates conservation between the two jaws in expression of the candidate reference genes, which might be required for maintaining the basic functions of the cells in skeletal tissues across all the species at the end of larval development. However, we observed more expression variations for all of these candidates in the pharyngeal jaws compared to the oral jaws suggesting more divergence in gene transcription in the pharyngeal jaws at the end of the larval phase (Fig. 1b and standard deviations in Table 2). According to BestKeeper ranking, *rpl18* and *tmem59* had respectively first and second least expression variations (lowest standard deviations) among the candidate reference genes in both jaws (Table 2). NormFinder ranked *rpl18* and *ube2l3* as the first and second most stably expressed reference genes in both jaws whereas geNorm ranked *ssrp1* and *rpl18* in oral jaw and *abcf1* and *rpl18* in pharyngeal jaw as the first and second best reference genes, respectively (Table 2). These observations demonstrated that out of the 12 candidates only *rpl18* was consistently ranked among the top two genes across the three rakings in both jaws. Therefore, we used the Cq value of *rpl18* in each sample as normalization factor (NF) for relative gene expression analyses of our target genes.

**Table 2:** Ranking and statistical analyses of reference genes in oral and pharyngeal jaws across all of the haplochromine species from three East African lakes

	Oral jaw						Pharyngeal jaw					
	BestKeeper		geNorm		NormFinder		BestKeeper		geNorm		NormFinder	
	Ranks	SD	Ranks	M	Ranks	SV	Ranks	SD	Ranks	M	Ranks	SV
1	*rpl18*	0.581	*ssrp1*	0.605	*rpl18*	0.272	*rpl18*	0.654	*abcf1*	0.619	*rpl18*	0.321
2	*tmem59*	0.639	*rpl18*	0.615	*ube2l3*	0.324	*tmem59*	0.813	*rpl18*	0.631	*ube2l3*	0.322
3	*pdia6*	0.640	*abcf1*	0.632	*abcf1*	0.376	*actb1*	0.984	*ube2l3*	0.633	*ssrp1*	0.331
4	*ube2l3*	0.649	*pdia6*	0.678	*pdia6*	0.398	*twf1*	1.016	*actb1*	0.672	*actb1*	0.412
5	*actb1*	0.663	*twf1*	0.683	*twf1*	0.424	*rps18*	1.026	*pdia6*	0.710	*pdia6*	0.442
6	*twf1*	0.688	*rps18*	0.687	*actb1*	0.448	*pdia6*	1.101	*rps18*	0.717	*abce1*	0.472
7	*ssrp1*	0.695	*actb1*	0.691	*rps18*	0.467	*ssrp1*	1.106	*twf1*	0.755	*twf1*	0.477
8	*rps18*	0.700	*abce1*	0.767	*ssrp1*	0.478	*abcf1*	1.106	*ssrp1*	0.764	*abcf1*	0.480
9	*abcf1*	0.717	*ube2l3*	0.773	*abce1*	0.513	*ube2l3*	1.150	*abce1*	0.769	*rps18*	0.517
10	*abce1*	0.720	*tmem59*	0.806	*tmem59*	0.587	*abce1*	1.151	*tmem59*	0.785	*tmem59*	0.577
11	*tbp*	1.031	*tbp*	0.900	*tbp*	0.639	*tbp*	1.328	*tbp*	0.896	*tbp*	0.665
12	*elf1a*	1.717	*elf1a*	1.579	*elf1a*	1.324	*elf1a*	2.162	*elf1a*	1.558	*elf1a*	1.257

SD indicates a ranking calculation based on standard deviation generated by BestKeeper, whereas SV, stability value, and M, mean expression stability value, are calculated by geNorm and NormFinder, respectively. In all of the ranking methods lower values represent more stably expressed reference genes

### Oral versus pharyngeal jaws expression differences of target genes in distinct trophic niches

**Fig. 2:**
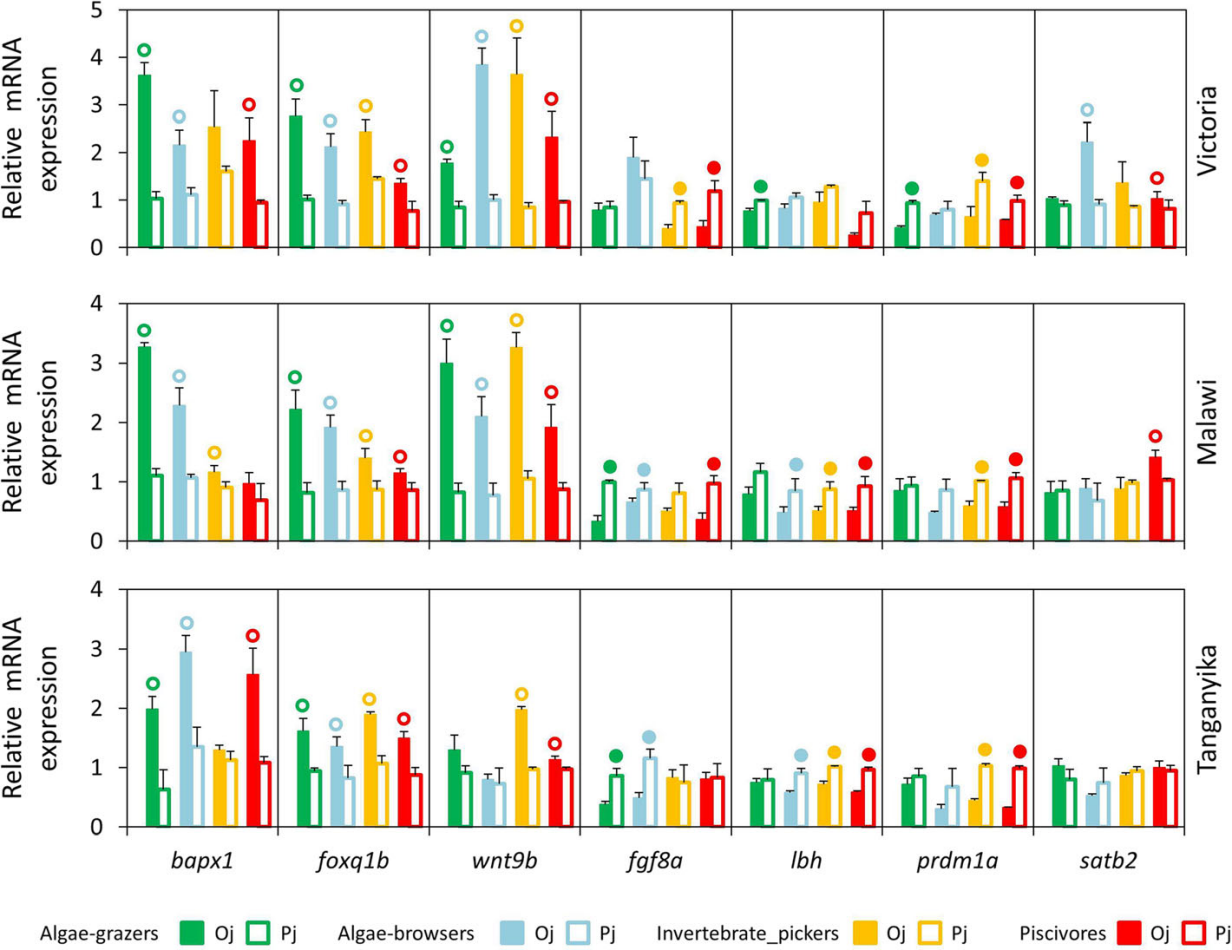
The oral versus pharyngeal jaws expression differences of seven target genes in haplochromine cichlids from three East African lakes at the end of larval phase. (A) Comparisons of relative expression levels between oral and pharyngeal jaws for seven candidate target genes in different lakes in East Africa at the yolk sac absorption stage marking the end of larval development and the onset of juvenile phase. Circles above bars indicate significantly elevated expression (*P* < 0.05) in comparisons between oral and pharyngeal jaws (i.e., compared to the bar matching the colour code of the circle); the comparisons were restricted within the species

All the seven candidate target genes had detectable expression levels (< 34 Cq values) in the oral and pharyngeal jaws, but their expressions were quite variable, from *foxq1b* with highest expression to *fgf8a* with lowest expression in both jaws (Additional file 2). The relative expressions of seven candidate target genes, *bapx1*, *foxq1b*, *wnt9b*, *fgf8a*, *lbh*, *prdm1a* and *satb2*, were compared between oral and pharyngeal jaws in each of the haplochromine species (Fig. 2). These jaw-specific comparisons revealed that all genes had tendency towards higher expression in only one jaw type, across all species and the three lakes. In other words, none of the genes showed higher expression in two different jaws across species and lakes. Among the seven candidate targets, four genes, *bapx1*, *foxq1b*, *wnt9b* and *satb2*, showed higher expression in the oral jaw (Fig. 2). The most consistent differential expression was observed for *foxq1b* which had higher oral jaw expression in all of the species from the three lakes indicating its conserved jaw specific transcriptional requirement. A similar differential expression pattern was found for *wnt9b* in all species from LM and LV, whereas only the two carnivorous species in LT showed higher oral jaw expression. The invertebrate-picker species across the three lakes showed slight or no expression difference for *bapx1* gene. The three other genes, *fgf8a*, *lbh* and *prdm1a* showed higher expression levels in the pharyngeal jaws than the oral jaws in several species. Interestingly, *prdm1a*, showed consistently higher pharyngeal jaw expressions in the carnivore species across the lakes suggesting its potential role in trophic niche-related morphological divergence of pharyngeal jaws at the end of larval development in haplochromine cichlids (Fig. 2). Although in an opposite manner, distinct carnivore-herbivore expression patterns were observed for *fgf8a* gene in LV and LT species, i.e. higher pharyngeal jaw expressions in carnivore and herbivore species in LV and LT, respectively. For *lbh* gene, the algae-grazer species displayed distinct differential expression patterns compared to other trophic niches across the lakes; the algae-grazers were the only species that did not show higher pharyngeal jaw expression of *lbh* in LM and LT, in contrast, the algae- grazer in LV was the only species showing higher *lbh* pharyngeal jaw expression (Fig. 2). Taken together, these observations demonstrate jaw-specific expression of the candidate target genes already at the late larval developmental stage (stage 26) prior to the onset of independent feeding. Also, some of these differences appear to arise from the parallel evolution of distinct trophic niches in the three lakes.

### Within jaw expression differences of target genes in distinct trophic niches

**Fig. 3:**
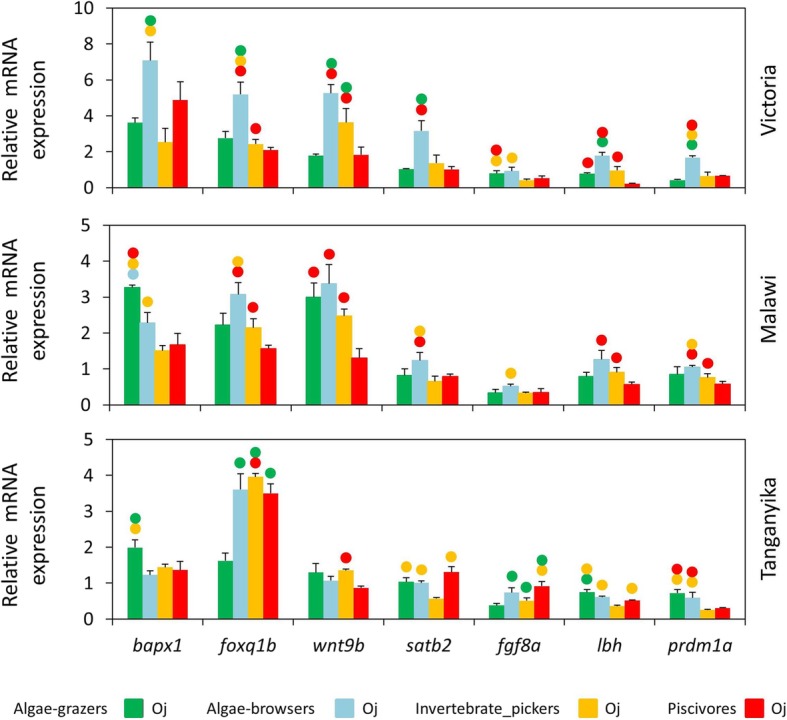
Oral jaws expression differences of seven target genes between distinct trophic niches in each lake at the end of larval phase. (A) Comparisons of relative expression levels of seven target genes between oral jaws of haplochromine species belonging to distinct trophic niche in each East African lake at the yolk sac absorption stage marking the end of larval development and the onset of juvenile phase. Circles above bars indicate significantly elevated expression (*P* < 0.05) in comparisons between oral jaws (i.e., compared to the bar matching the colour code of the circle)

**Fig. 4:**
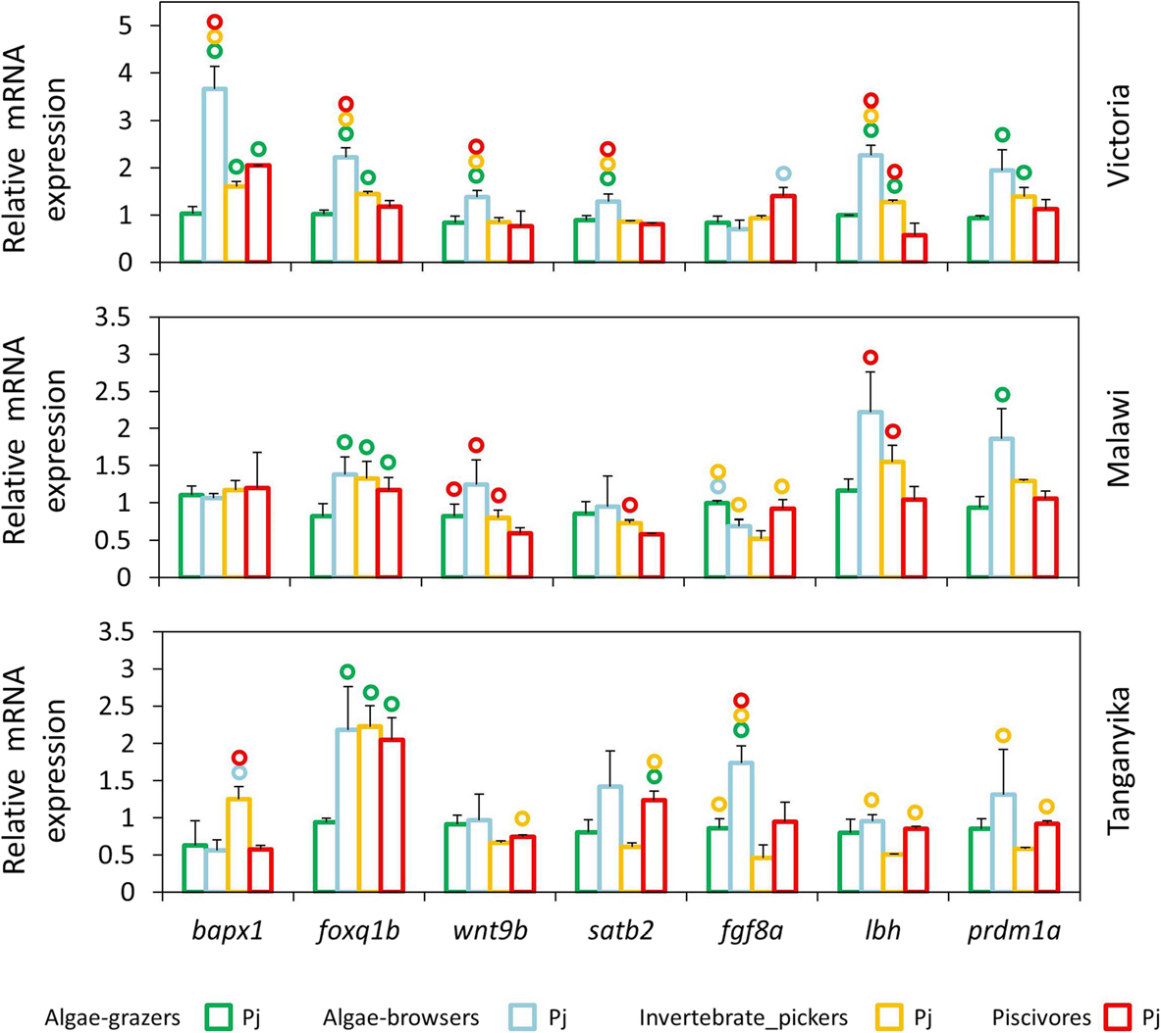
Pharyngeal jaws expression differences of seven target genes between distinct trophic niches in each lake at the end of larval phase. (A) Comparisons of relative expression levels of seven target genes between pharyngeal jaws of haplochromine species belonging to distinct trophic niche in each East African lake at the yolk sac absorption stage marking the end of larval development and the onset of juvenile phase. Circles above bars indicate significantly elevated expression (*P* < 0.05) in comparisons between pharyngeal jaws (i.e., compared to the bar matching the colour code of the circle)

Next, we compared the expression of the target genes between the species of each lake in oral or pharyngeal jaws (Figs. 3 and 4). In oral jaws, we did not find consistent herbivore-carnivore expression differences across the lakes, however, we found some other similarities in trophic niche-based expression differences (Fig. 3). For instance, the algae-browser species in LV and to a lesser extent in LM displayed higher oral jaw expression for almost all the target genes compared to other trophic niches. In addition, relatively similar differential expression patterns between the trophic niches were observed for *foxq1b*, *satb2* and *lbh* genes in LM and LV. The only gene with clear herbivore-carnivore expression differentiation was *prdm1a* in LT indicating that none of the selected target genes can act as differentiating factors in herbivore versus carnivore skeletal morphogenesis in the oral jaw at the end of larval development (Fig. 3). In the pharyngeal jaw, on the other hand, we again found higher expression of almost all target genes (except *fgf8a*) in the algae-browser species of LV but such tendency was not observed in the two other lakes (Fig. 4). Moreover, differential expression patterns between the trophic niches for three genes, *wnt9b*, *lbh* and *prdm1a* showed similarities in LM and LV. The most striking pattern was observed for *foxq1b*, which showed lower expression in the algae-grazer species in all the lakes compared to other species with different trophic niches (Fig. 4). This could imply on the involvement of *foxq1b* in parallel evolution of the specialized pharyngeal jaw morphology of the algae-grazer species in the three lakes.

### Expression correlation analyses of target genes in oral and pharyngeal jaws

Finally, we were interested to investigate potential modularity in transcriptional regulations of the candidate target genes between the oral and pharyngeal jaws at the end of larval development which could also differ across the lakes or herbivore-carnivore trophic specialization. To do this, we first analysed expression correlations of the target genes in the two jaw types using species of each lake (Table 3). Our expectation was that lower correlations would indicate evolutionary modularity in gene expression between the two jaws and high correlation would indicate low modularity and high integration. Interestingly, we found expression correlations between oral and pharyngeal jaws in six out of seven target genes in LV whereas only two genes appeared to have such expression correlations in LM and LT. This suggests a smaller degree of modular transcriptional regulation of the target genes between the two jaws in the species of the youngest lake (LV) compared to the other lakes at the end of larval phase, raising the possibility of increasing transcriptional modularity in feeding structures with increasing divergence and thus evolutionary age. It should be noted that *fgf8a* showed no expression correlations between the two jaws in all the lakes, and on the contrary, *lbh* displayed such correlations in all the lakes (Table 3). In addition, we also explored expression correlations between the two jaws based on trophic specialization by classifying the herbivore and carnivore species from the three lakes into two distinct groups (Table 3). We again did not find any correlation between the two jaws for *fgf8a* expression whereas positive expression correlations between the jaws were observed for *lbh* and *foxq1b* in both herbivores and carnivores. Furthermore, we found *satb2* showing positive expression correlation between the two jaws only in carnivores, while *bapx1* and *prdm1a* had positive expression correlations between the two jaws in herbivores. These observations imply a distinct modular expression of the target genes between the contrasting trophic structures at the end of larval development and prior to onset of juvenile phase and feeding in East African haplochromine cichlids.

**Table 3:** The lake- and trophic niche-based analyses of expression correlations between oral and pharyngeal jaws for seven target genes in the haplochromine cichlids at the end of larval phase

	Oral versus pharyngeal jaws				
Gene	Lake Victoria	Lake Malawi	Lake Tanganyika	Herbivores	Carnivores
*bapx1*	*r* = 0.801 *P* < 0.01**	NS	NS	*r* = 0.827 *P* < 0.001***	NS
*foxq1b*	*r* = 803 *P* < 0.01**	NS	*r* = 0.889 *P* < 0.001***	*r* = 0.793 *P* < 0.001***	*r* = 0.707 *P* < 0.01**
*wnt9b*	*r* = 0.792 *P* < 0.01**	*r* = 0.744 *P* < 0.01**	NS	NS	NS
*satb2*	*r* = 0.79 *P* < 0.01**	NS	NS	NS	*r* = 0.798 *P* < 0.001***
*fgf8a*	NS	NS	NS	NS	NS
*lbh*	*r* = 0.962 *P* < 0.001***	*r* = 0.891 *P* < 0.001***	*r* = 0.62 *P* < 0.05*	*r* = 0.835 *P* < 0.001***	*r* = 0.897 *P* < 0.001***
*prdm1a*	*r* = 0.837 *P* < 0.001***	NS	NS	*r* = 0.613 *P* < 0.01**	NS

Pearson correlation coefficient (r) was used to assess the similarity between expression pattern of the target genes between oral and pharyngeal jaws across the haplochromine species in each lake or trophic niche specialization. NS indicates no significant expression correlation between the two jaw types in a given comparison

## Discussion

Unraveling the molecular and transcriptional basis of divergent eco-morphologies is an important step towards understanding how such traits arise and evolve. The two modular jaws of cichlid fishes adapted to different trophic niches in parallel radiations present an excellent comparative framework to explore the expression of genes underlying this speciation trait. This study to our knowledge is the first to explore gene expression at the end of larval stage in both the oral and pharyngeal jaws in divergent and parallel trophic morphologies. We believe that targeting a developmental stage that marks the end of larval development, before the young juveniles forage for the first time, is a critical moment to mark the transcriptional trajectory that is ready to respond to external stimuli. To explore this, we selected a set of candidate genes with modular expression pattern in skeletal structures of teleost fish related to feeding and with known functions in skeletal morphogenesis during embryonic stages (Table 1).

One of the candidate target genes of paramount importance in our study was *lbh* encoding a transcription cofactor which is evolutionary conserved across vertebrates [48]. The function of *lbh* is mainly known as a modulator of cell cycle progress and for its involvement in early limb and heart development [36, 49, 50], embryonic angiogenesis and endochondral bone formation [51], as well as regulation of photoreceptor differentiation in zebrafish [37]. It has been shown that *lbh* can activate MAPK pathway through promoting transcriptional activation of Ap-1 complex (fos/jun heterodimer) [50], and notably, different components of the same pathway are known to play a role in the morphogenesis of trophic skeletal structures in teleost fishes [38]. Moreover, transcriptional changes in a major component of Ap-1 complex (*fos*) is also implicated in adaptive phenotypic plasticity of pharyngeal jaw in response to mechanical strains in cichlid [17, 19]. This raises the possibility that differential expression of *lbh* affects jaw skeletal morphogenesis in a similar manner as plastic mechanical responses exert effects on jaw morphogenesis through transcriptional regulation of Ap-1 complex. The reason for selecting *lbh* as a target gene in our study was based on the fact that it is the only identified gene with a polymorphism associated with continuous morphological variation in the cichlid jaw and its function during jaw morphogenesis is characterized [52]. The differential expression of *lbh* along anterior-posterior arches at early craniofacial patterning seems to be a determinant of jaw morphological variations in later stages of development in cichlids [52]. In our study, we found a tendency for higher *lbh* expression in pharyngeal jaw than oral jaw at the end of larval development, and the algae-grazer species in each lake appeared to have different jaw-specific *lbh* expression compared to the other trophic niches. Also, *lbh* displayed fairly similar differential expression pattern between the contrasting trophic niches in both jaws of LM and LV species. This suggests potential role of *lbh* in divergent jaw morphogenesis long after early developmental patterning of craniofacial skeleton. Interestingly, *lbh* was the only gene among our candidates showing expression correlation between the two jaws in different comparisons (i.e. lake- and trophic-based comparisons) suggesting its non-modular transcriptional regulation along anterior-posterior feeding skeletal structures. It is already known that *lbh* can be itself a downstream target of Wnt signaling [53], a key signaling pathway involved in various aspects of developmental patterning, morphogenesis and growth of trophic skeletal structures in fish [38]. Differential regulation of Wnt signaling pathway is already suggested as a major player in emergence of craniofacial phenotypic variations in African cichlids [23, 54, 55].

Perhaps the most striking finding of our study was the expression pattern of *prdm1a* encoding a transcriptional activator and repressor regulating neural crest development in zebrafish embryos [24], and selected as a target in our study because of its critical role in development and morphogenesis of posterior trophic skeletal structures [25]. *prdm1a* is a direct downstream effector of retinoic acid (RA) signalling pathways [25], which is the pivotal pathway in development and morphogenesis of skeletal derivatives of posterior pharyngeal arches [26, 38]. We found *prdm1a* to have consistently higher expression in the pharyngeal jaw than the oral jaw in carnivore species of the three lakes at the end of larval phase indicating its potential role in adaptive divergence of posterior trophic skeleton in haplochromine cichlids at later stages of development. Interestingly, a correlation in *prdm1a* expression was observed between the two jaws only in herbivore species which could implicate a transcriptional regulatory decoupling of *prdm1a* between the two jaws in carnivore species. It is worthy to further investigate whether receiving different diets during juvenile phase can influence the distinct herbivore-carnivore expression pattern of *prdm1a* in cichlid jaws. In particular, *prdm1a* might be a mediator of the effects of activated RA through different nutritional conditions on trophic skeletogenesis in later stages of development such as the early feeding period [27].

We tested expression of three target genes, *bapx1*, *foxq1b* and *wnt9b* with a specific role in the formation of oral jaw skeletal elements. The first gene, *bapx1*, encodes a member of the NK family of homeobox-containing proteins and plays an important role in positional specification of the oral jaw joint and its articulation in gnathostomes [28–30]. The activity of *bapx1* is required for morphogenesis of the retroarticular process and mandible and its expression is controlled by activity of FGF, BMP and Endothelin signaling pathways [29, 30]. The specialized modes of oral jaw feeding structures originate from the opening and closing capabilities of the lower oral jaw in contrasting trophic niches of cichlids. These capabilities depend on distinct morphologies of the lower jaw and retroarticular process together with the position of the jaw joint, and hence, the role of genes like *bapx1* would be of paramount importance for investigations of the molecular basis underlying divergent jaw morphogenesis [31, 32]. In our study, we found higher expression of *bapx1* in the oral jaw than pharyngeal jaw of most species, but such difference was either not observed or not pronounced in the invertebrate-picker species. Furthermore, among LM species, the herbivore species had markedly higher oral jaw expression of *bapx1* compared to the carnivores. The oral and pharyngeal expression of *bapx1* was found to be correlated in herbivore species across the three lakes whereas such correlation was not observed in carnivores. These observations suggest a potential role of *bapx1* in divergent morphogenesis of oral jaw skeleton in later stages of development in cichlids.

The second gene, *foxq1b*, encodes a member of Forkhead-Box transcription factor family that is known to have a specific developmental expression pattern confined to the oral jaw in zebrafish [33, 34]. *foxq1b* plays a role in the formation of the lower jaw, particularly Meckel’s cartilage and associated structures, and it is a major mediator of the effects of Aryl hydrocarbon receptor (AHR) pathway in early patterning and later larval stages of jaw development in zebrafish [33]. The AHR pathway is not only the key mediator of the developmental effects of various environmental compounds on jaw skeletogenesis but has also regulatory cross-talks with several other critical signaling pathways during skeletogenesis, such as Wnt, Hedgehog and Ca2+/Calmodulin pathways [38], which play an important role in adaptive divergence of cichlid craniofacial structures [18, 35, 39, 54, 55]. The AHR pathway is also demonstrated to have intrinsic developmental role in elongation of oral jaw structure in zebrafish [40] and differential expression of its components including *foxq1b* is shown to be associated with morphological divergence of jaw skeleton during larval development in Arctic charr, a salmonid species [41]. In our study, we found higher oral jaw expression of *foxq1b* in all species at the end of larval development suggesting its conserved role in oral jaw formation in teleost fish. We also found lower expression of *foxq1b* in the pharyngeal jaw of algae-grazer species compared to other trophic niches indicating its potential role in morphological divergence of the pharyngeal jaw in haplochromine cichlids. The *foxq1b* expression also showed a correlation between the pharyngeal and oral jaws in both herbivorous and carnivorous species, which proposes shared transcriptional regulatory mechanism at a late stage of larval development.

The third gene, *wnt9b*, is a member of the WNT gene family encoding a secreted signaling protein required for dorso-ventral patterning of oral jaw and outgrowth of upper jaw in zebrafish [42, 56]. Expectedly, we found higher oral jaw expression of *wnt9b* in all species except the herbivorous species from LT, also, more similar expression patterns for *wnt9b* were found in the jaws of species from LM and LV. These observations could reflect more diverged *wnt9b* transcription in LT haplochromine cichlids compared to the two other lakes.

In addition to the abovementioned candidates, we tested the expression of two other genes, *fgf8a*, a member of the fibroblast growth factor (FGF) family, and *satb2*, a DNA binding protein which binds to nuclear matrix acting as a multifunctional determinant of craniofacial patterning and osteoblast differentiation during development [57]. Activation of FGF signaling is required for the formation of almost all craniofacial skeletal structures in different developmental stages of vertebrates [38, 58]. More specifically, *fgf8a* expression in pharyngeal pouches is required for migrating pouch-forming cells towards mesodermal guideposts, a crucial mechanism in viscerocranial skeletogenesis [59]. In later stages of jaw development, expression of *fgf8* (together with other FGF family members) is a determinant of skeletal size in posterior pharyngeal arches [60, 61]. In our study, we found higher pharyngeal than oral jaw expression of *fgf8a* at the late larval stage 26. However, this pattern was not consistent across the three lakes and even an opposite expression pattern concerning trophic niche was observed between LM and LT. Importantly, *fgf8a* was the only gene showing no expression correlation between the two jaws in the different comparisons, indicating that its regulatory decoupling along the anterior-posterior axis might be associated with the emergence of the pharyngeal jaw in cichlids. Finally, *satb2*, was the only gene showing expression correlation between the two jaws specifically in carnivores at the late larval stage, indicating its potential role in distinct and modular morphogenesis of the two jaw types in the herbivore cichlids. *satb2* gene has been shown to have high degree of conservation in gnathostomes [62] and its developmental expression determines jaw length in a modular manner (for instance by affecting size of distal jaw module), and moreover, its expression variations found to be associated with evolvability of the distal jaw domain [63].

The morphology of cichlid jaws has evolved repeatedly, along similar morphological trajectories in Lake Tanganyika, Malawi, and Victoria. The cichlid radiations in these lakes essentially represent the same process of evolutionary diversification, but at different stages linked to their age [64, 65]. The age of the lakes is also associated with the extent of morphological modularity exhibited in traits. It was previously shown using geometric morphometric analysis that the head morphology of cichlid fishes from the youngest Lake Victoria was the most integrated (least modular), followed by older Lake Malawi and much older Lake Tanganyika. Interestingly, we observed a similar pattern in the gene expression correlations of the seven target genes across lakes, with the lowest number of modular genes in Lake Victoria compared to the two older lakes (Table 3). Thus, for the first time we provide some evidence of the link between decoupling of gene expression in cichlid jaws and the evolutionary age of the cichlid radiations.

## Conclusions

This is the first attempt to study modularity in gene expression between cichlid oral and pharyngeal jaws simultaneously, across diverse trophic niches in parallel adaptive radiations. Our results provide evidence of distinct modular expression of key genes involved in jaw morphogenesis in relation to trophic niche specialization prior to the onset of independent feeding in cichlid larvae. We also show that the gene expressions of the jaws in cichlids from the younger lake are less modular, consistent with previous studies on their morphological integration. Our findings shed light on the molecular and transcriptional organization of the oral and pharyngeal jaws at the end of postembryonic development, in anticipation of environmental stimuli. The expression divergence prior to plastic response to feeding for modularly-regulated genes and the differences between the two jaw types might contribute to pre-feeding trophic canalization in cichlids, and therefore, requires further investigations. This initial exploration can be expanded upon by adding more genes or even whole transcriptomes to unravel transcriptional basis of cichlid jaw modularity and parallelism. Another interesting example for a future study could be the genes involved in bone-remodeling, as it has been already observed in other teleost fish that their differential expression during early and late developmental stages (prior to foraging) could differentiate between contrasting trophic jaw morphologies [66, 67].

## Methods

### Fish husbandry and sampling

Twelve haplochromine cichlid species from Lakes Victoria, Malawi, and Tanganyika, which are adapted to two major trophic niches via convergent evolution, were selected for this study (4 species each, see Fig. 1a). In each lake, two herbivorous species (an algae-grazer and an algae-browser) and two carnivorous species (an invertebrate-picker and a piscivore were selected) for comparisons of trophic niche specializations (Fig. 1a) [68, 69]. Carnivores have a front-oriented mouth and predominately unicuspid teeth. Algae grazers have a large and slightly downwards oriented mouth with long comb-like bi- and tricuspid teeth, while algae browsers have bi- and tricuspid teeth, plus a different head shape with a downwards oriented mouth. The fish were raised in standardized tanks and rearing conditions and on the same diet (Spirulina flakes) until mating behaviour was observed. After the spawning period, up to four hours depending on species, the eggs were removed from the mouth of the females by inserting slight manual pressure on their cheeks. The eggs were incubated under very gentle shaking in small standard glass jars with 20 cm diameter. The hatched larvae were transferred to larger tanks until the end of larval development, defined as stage 26 in cichlids [21, 70]. Six larvae per species were sacrificed by euthanization in water with 0.2 g MS-222/l, and their oral and pharyngeal jaws carefully dissected under the stereomicroscope. Tissues from two individual oral or pharyngeal jaw samples per species were pooled to represent one biological replicate, and three biological replicates per species were used for gene expression analysis in this study. The parents of the twelve haplochromine species were also euthanized in water with 0.8 g MS-222/l at the end of the study.

### RNA isolation and cDNA synthesis

For the RNA isolation two dissected oral or pharyngeal jaws per replicate were pooled into a single tube with 0.25 mL of a tissue lysis buffer provided by Reliaprep RNA tissue miniprep system (Promega, #Z6111, USA) together with one 1.4 mm ceramic sphere to crush the jaws. The jaws were homogenized using a FastPrep-24 Instrument (MP Biomedicals, Santa Ana, CA, USA) and RNA was isolated based on the instructions by the manufacturer adjusted for small amounts of fibrous tissue. In brief, the instruction includes the mixing of the lysis buffer and homogenized tissue with isopropanol and passing it through a column provided by the kit, several RNA washing steps and an in-column DNase treatment. The quantity of RNA was checked using a Nanophotometer (IMPLEN GmbH, Munich, Germany) and the quality was assessed with RNA ScreenTapes on an Agilent 2200 TapeStation (Agilent Technologies). The RNA samples with a RNA integrity number above seven were subjected to first strand cDNA synthesis using 700 ng of RNA and High Capacity cDNA Reverse Transcription kit (Applied Biosystems). The resulting cDNA of each sample was diluted 1:10 times in nuclease-free water to be used for qPCR steps.

### Selection of candidate genes and primer design

We selected seven candidate reference genes with the highest expression levels in transcriptome data of the jaws from LT Haplochromine cichlids which also had shown no significant expression differences between the two jaw types and across species at stage 26 [16]. Furthermore, we added five more reference genes that were previously validated in qPCR based studies on African cichlids [19, 44–47, 71, 72]. As target candidates we selected seven genes, including *bapx1*, *foxq1b*, *wnt9b*, *fgf8a*, *lbh*, *prdm1a* and *satb2*, involved in modular morphogenesis of trophic skeletal system along the anterior-posterior axis in teleost fishes (described in the discussion section). The primers were designed at conserved sequence regions using the available transcriptomes of five East African haplochromine species (*Pundamilia nyererei*, *Simochromis diagramma*, *Gnathochromis pfefferi*, *Metriaclima zebra*, and *Astatotilapia burtoni*) and two more distantly related cichlid species (*Oreochromis niloticus* and *Neolamprologus brichardi*) [16, 73]. The coding sequences of all species were aligned in CLC Genomic Workbench, version 7.5 (CLC Bio, Aarhus, Denmark) and exon boundaries were delineated using the *Oreochromis niloticus* annotated genome in the Ensembl database (http://www.ensembl.org) [74]. The primers were designed over these boundaries with a short amplicon size (< 250 bp) suitable for qPCR quantification [75]. Primer Express 3.0 (Applied Biosystems, CA, USA) and OligoAnalyzer 3.1 (Integrated DNA Technology) were used to design the primers with minimal occurrence of dimerization and secondary structures.

### qPCR and data analysis

The instruction suggested by Maxima SYBR Green/ROX qPCR Master Mix (2X) (Thermo Fisher Scientific, Germany) was used to produce qPCR reactions. The amplification steps were performed in 96 well-PCR plates through ABI 7500 real-time PCR System (Applied Biosystems). For each biological replicate two technical replicates were assigned and we followed an approved experimental set-up known as sample maximization method to obtain to optimal qPCR conditions [76]. The qPCR program and a dissociation step were performed as described in a previous gene expression study of cichlid [77], and primer efficiencies were determined by LinRegPCR v11.0 programme (http://LinRegPCR.nl) [78] (Additional file 1).

Three different methods were utilized to validate the most stable reference genes including BestKeeper [79], NormFinder [80] and geNorm [81], which in turn take into account the lowest standard deviations (SD) of raw quantitation cycle values (Cq), mean values (M) and stability values (SV) to rank most suitable reference genes. The Cq value of the most stable reference gene was used as normalization factor (Cq _reference_), and then ΔCq of each target gene was calculated (ΔCq _target_ = Cq _target_ – Cq _reference_). In expression comparisons within the jaw types for each target gene a pharyngeal jaw replicate of an algae-grazer species in each lake was used as a calibrator sample and rest of the samples were normalized to its ΔCq value to calculate ΔΔCq values (ΔCq _target_ – ΔCq _calibrator_). In expression comparisons between the jaw types for each target gene a pharyngeal jaw replicate for each species was used as a calibrator sample. Relative expression quantities were calculated for the normalized values using E^−ΔΔCq^ [82] and then fold difference values were calculated by transformation of RQ values to logarithmic base 2 values in order to conduct further statistical analysis [83]. The significant expression differences were determined using ANOVA statistical tests, followed by Tukey’s HSD post hoc tests. The lake- or trophic-based expression correlations between the jaw types were calculated through Pearson correlation coefficients (r) for each gene using R (http://www.r-project.org).

## Additional files

Additional file 1: Information about qPCR primers used in this study. (XLSX 12 kb)

Additional file 2: Statistical results and raw gene expression data. (XLSX 31 kb)

## Data Availability

All data generated or analysed during this study are included in this published article.

## Abbreviations

*bapx1*: Bagpipe homeobox protein homolog 1
*fgf8a*: Fibroblast growth factor 8a
*foxq1b*: Forkhead box protein q1b
*lbh*: Limb bud and heart development
LM: Lake Malawi
LT: Lake Tanganyika
LV: Lake Victoria
*prdm1a*: Positive regulatory domain I-binding factor 1
*satb2*: Special AT-rich sequence-binding protein 2
*wnt9b*: Wingless-type MMTV integration site family 9b
